# Uncommon Etiology for Seizure: Cerebral Hyperperfusion Syndrome

**DOI:** 10.1155/2017/7965758

**Published:** 2017-05-10

**Authors:** Mohankumar Kurukumbi, Ahn Truong, Naghemeh Pirsaharkhiz

**Affiliations:** ^1^Department of Neurology, Inova Fairfax Hospital, Falls Church, VA, USA; ^2^VCU School of Medicine, Inova Campus, Falls Church, VA, USA; ^3^Department of Neurology, VCU School of Medicine, Inova Campus, Falls Church, VA, USA

## Abstract

Cerebral hyperperfusion syndrome (CHS) is a rare life-threatening complication of carotid endarterectomy (CEA) and carotid artery stenting (CAS) for carotid artery stenosis. The incidence varies between 0 and 3%, depending on the severity of the stenosis, perioperative hypertension, and contralateral carotid stenosis. This case report reports a 53-year-old female patient presenting with decreased alertness and multiple tonic-clonic seizures, in the background of bilateral CEA. She was found to have bilateral carotid stenosis. Her left CEA was performed three months prior and right CEA was four days prior to her current presentation with seizures. After bilateral CEA, the imaging showed extensive pathologic process involving primarily the subcortical white matter and overlying cortex, more on the right cerebral hemisphere. On follow-up six weeks later, she reported no recurrent seizures and imaging showed decrease in abnormal signal intensity of the grey and white matter. This was indicative of near complete resolution of hyperperfusion damage. CHS is a rare complication due to the loss of autoregulation of the cerebrovascular system and increased blood flow status after bilateral CEA. This case is reported because of a rare and unique presentation of seizures in the background of bilateral CEA.

## 1. Introduction

Cerebral hyperperfusion syndrome (CHS) is a rare complication of carotid surgery that is described as an increase of cerebral blood flow >100% compared to baseline that leads to acute headache ipsilateral to carotid revascularization, disorientation, focal neurological deficits, and seizures [1]. Here, we present a rare case of CHS, complicated by multiple grand mal seizures and intracranial hemorrhage.

## 2. Case

**Figure 1:**
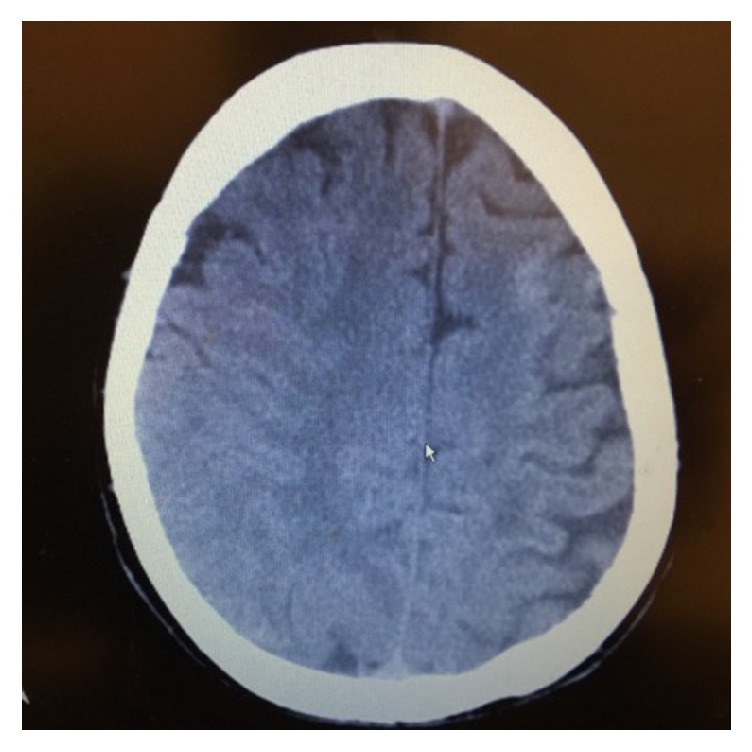
CT head showing left frontal hyperdense lesion on left frontal region.

**Figure 2:**
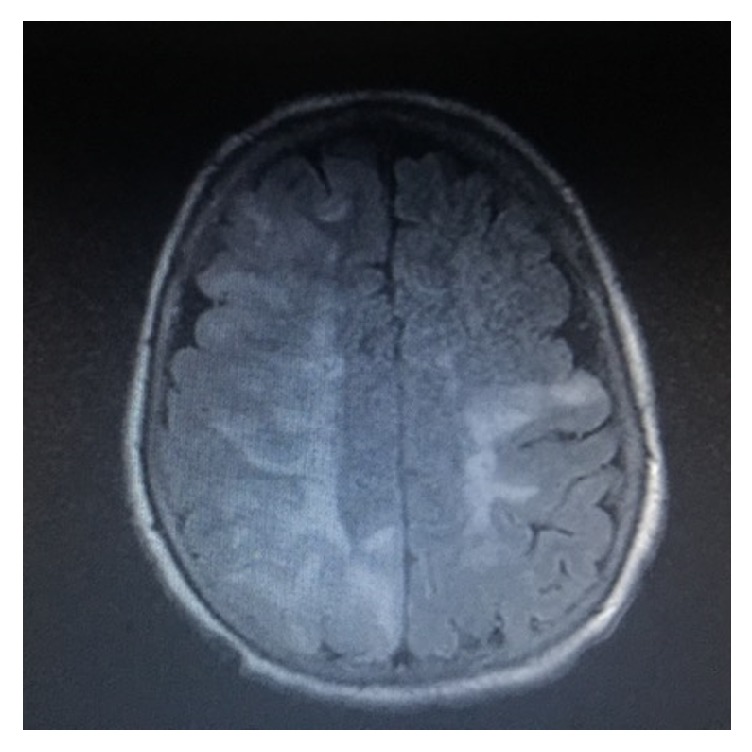
Initial MRI brain (FLAIR) showing bilateral frontal hyperintense white matter lesions, right > left.

A 53-year-old Caucasian female with past medical history of insulin dependent diabetes mellitus, hypertension, hyperlipidemia, stroke with no residual deficits, and significant bilateral carotid stenosis status after endarterectomy presented to the emergency department, four days after her right carotid endarterectomy (CEA) with acute onset of headache, confusion, and witnessed seizures enroute to the hospital. Patient had left sided CEA two months priorly with no complications. On the day of presentation, she was in her usual state of health until suddenly she was found with decreased alertness and complaining of headache. She had two tonic-clonic seizures in the ambulance, was intubated for airway protection, and was transferred to critical care unit and started on antiepileptics. Her condition was further complicated by septic shock secondary to aspiration pneumonia. Patient was put on continuous electroencephalogram monitoring which showed diffuse slowing compatible with an encephalopathic picture with no clear epileptiform activity. On physical exam, BP of 174/75 was documented and she exhibited left sided facial droop, dysarthria, and mild left sided gaze palsy, as well as mild weakness in right upper and right lower extremities. CT (Figure 1) indicated left frontal increased attenuation and MRI (Figure 2) extensive pathologic process involving primarily the subcortical white matter and overlying cortex of throughout both cerebral hemispheres, primarily bilateral frontal lobe lesions (right > left). Luxury perfusion of an ischemic area is considered as possible etiology.

**Figure 3:**
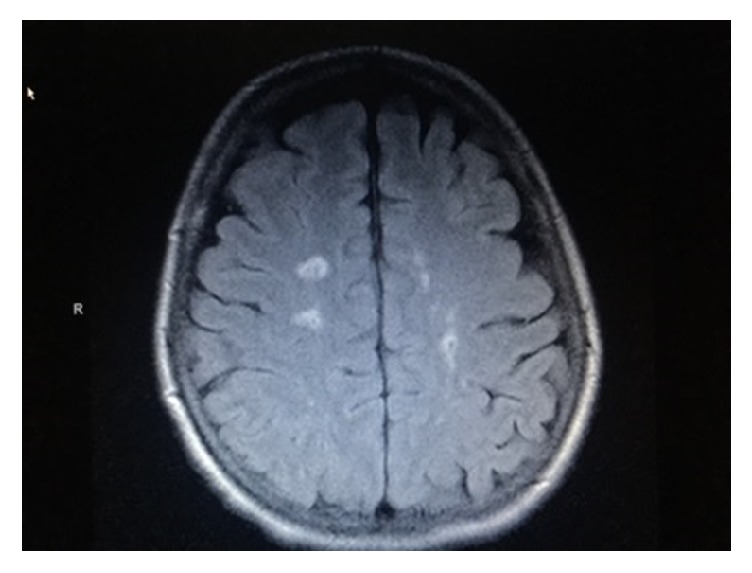
Six-week follow-up MRI brain (FLAIR) showing improvement in bilateral hyperintense lesions.

This patient's blood pressure was managed with Nimodipine for the duration of her hospital stay ranging from 140 to 160 systolic range, and she was later discharged on her usual Lisinopril for hypertension management. Her MRI (Figure 3) at the six-week follow-up reveals only minor residual edema and resolution of the hyperintense lesions. Her mental status, dysarthria, and limb weakness have improved back to baseline and she has not experienced further seizures, although she was kept on Levetiracetam for 6 months.

## 3. Discussion

Systematic analyses of numerous studies with large patient populations estimate the incidence of CHS after CEA or carotid artery stenosis (CAS) between 0.4% and 1.8% [2]. Risk factors predisposing patients to this condition include longstanding hypertension, prolonged or significant stenosis, poor collateral blood flow, and stenosis in the contralateral carotid artery, all of which were present in this patient.

Regulatory mechanisms of cerebral vasculature in a healthy individual are able to maintain a relatively constant cerebral blood flow with fluctuations of systemic blood pressure in the range of 60 to 160 mm Hg [3]. These mechanisms include the metabolic regulation affecting the arterioles and the myogenic response of the carotids. The stretch reflex of the carotids contracts the smooth muscles of the artery in response to increased blood pressure, thus decreasing the cerebral blood flow. In patients with carotid stenosis that consequently leads to chronic ischemic conditions, resistant arterioles are persistently dilated to maintain sufficient blood flow until the point of maximal dilation at rest [3]. The constant dilation eventually leads to damaging of the smooth muscles and inability of arterioles to dilate or constrict in response to stimuli [3]. Microangiopathic changes secondary to chronic hypertension and diabetes mellitus, both present in our patient, further complicate the impairment of regulatory mechanisms by causing thickening of the vessels and weakening the myogenic response. This paralyzed autoregulation after surgical correction of the high-grade stenosis thus leads to breakthrough perfusion pressure resulting in edema and hemorrhage [3, 4].

Characteristic clinical symptoms of CHS include severe unilateral headache, pain in face and eye, acute changes in mental status, seizure, and focal neurologic deficits related to cerebral edema or intracranial hemorrhage [5, 6]. Seizures are a relatively uncommon manifestation, seen in about 3% of hemodynamically compromised patients [7]. Cerebral edema caused by the hyperperfusion is the major cause of seizures [4].

MRI is the best tool to detect CHS at early stages for detection of edematous lesions. Single-photon emission computed tomography is usually used 48–72 hours after carotid revascularization as it is a sensitive method to measure cerebral blood flow and detect CHS.

Most CHS patients have a favorable prognosis with complete recovery with prompt diagnosis and treatment [8]. Delayed diagnosis or progression to intracranial hemorrhage is associated with poorer prognosis with up to 30% chronic partial disability and up to 50% mortality rate [8, 9].

Management of CHS patients is mainly done through prompt and rigorous blood pressure control via agents such as labetalol or clonidine to avoid cerebral vasodilation that is normally present with calcium channel blockers or nitrates [1]. The goal is for the systolic blood pressure to be <140 mmHg and treatment must be continued until cerebral hyperperfusion resolves and autoregulation is restored [1, 3]. In presence of significantly increased intracranial pressure secondary to cerebral edema, sedation and osmotic agents such as mannitol or hypertonic saline may be necessary. Anticonvulsant therapy is indicated for patients who develop seizures for a period of 3–6 months [1].

## 4. Conclusion

This case is reported due to the rare and unique presentation of CHS in this patient. Currently, there are no clear guidelines for repeat MRI after CAE and larger population studies are required to determine such guidelines. Unfortunately, due to rarity of CHS, most studies are limited by small sample sizes [3].

## Abbreviations

CHS: Cerebral hyperperfusion syndrome
CEA: Carotid endarterectomy
CT: Computed tomography
MRI: Magnetic resonance imaging
FLAIR: Fluid-attenuated inversion recovery
CAS: Carotid artery stenosis.
